# Supplementary material for: Patterns of Intron Gain and Loss in Fungi
Source: PLoS Biol. 2004 Nov 30;2(12):e422. doi: 10.1371/journal.pbio.0020422 (PMC532390; doi:10.1371/journal.pbio.0020422)
Supplement: Table S1 — Also available at http://genes.mit.edu/NielsenEtAl/. (4.3 MB ZIP). [file pbio.0020422.st001.zip › NielsenEtAl/html/124.html]

AN4417.1.NCU06776.1.MG03886.1.FG10865.1


```
 CLUSTAL W (1.82) Multiple Sequence Alignments - Introns Inserted


Sequence 1: NCU06776.1	632 aa
Sequence 2: MG03886.1	643 aa
Sequence 3: FG10865.1	620 aa
Sequence 4: AN4417.1	633 aa
Alignment Length: 680 aa
Number Identitical Residues: 114 aa
Alignment Score (without introns) 8526


MG03886.1 	MAPPTVRRAPYKDFLQPAFHRR~FSSTATVILAIAYLESVLLAKWNSY1LWTWFPIGPAG
NCU06776.1	MASASVRRPPYKDFLQPALHRR~FSTTALILLVIAYVYAIGLARWNSF1LWSWFPIGPTG
FG10865.1 	MAATTVRKSPYKDFLQPALHRR~FTSTATVLLIVSYLQAIILDSWSSY1FWAWFPIGPAG
AN4417.1  	---MAAKPRPYRRILTSALHRS2LSVFD-----------------LEV~FWALFPLGSCG
          	    :.:  **: :* .*:**  ::                     .  :*: **:*. *

MG03886.1 	IRTFFIFLCGLFVLILRIAQYHVGIRISNSGLETVQQHALKLQTLEAVVSYVLSSWLFSQ
NCU06776.1	FRAAFFWFSGSLILILRIAQYHPGYRTSDSAFDTFLKHWRNFSTLETILTYAFSAWLFAQ
FG10865.1 	FRTAFIFTCGLAIVVLRIANYHVGLRTTGSGFQTLQTTVTQLKTYETLFWYGFSSLLFSH
AN4417.1  	IRTILLFISPLAIFVLRVGQMHIGSRTTSSSLNTFHYLFP-LHVIQTFGWYIFSAWWFSE
          	:*: ::: .   :.:**:.: * * * :.*.::*.      : . ::.  * :*:  *:.

MG03886.1 	TYLGAVPSSANLDWITYFN~-GDRPRLNEKPIFFTVHFIILGIYQAVSHIYYDQDRLTLG
NCU06776.1	VYLLSQPKDSGLEWVTYLS~-YDRQRLNEKAVFITTHLVMLGVYQAIRHLYLDIDRLLLG
FG10865.1 	VFLWSMHSTANLSWITYFH~-GDRARLNERPLFLLAYMISCAITQTFNHYRRDTDRLVLG
AN4417.1  	LYKWSAPTSAHLEWVKRGS2PHERATLNERTIYIYTYHLLLAIAQSLAHLYNDYDRVPIA
          	 :  :  . : *.*:.    . :*  ***:.::: .: :  .: *:. *   * **: :.

MG03886.1 	VAKP-RKEGKAGAVDTGSQLKKFGEQLPAIFRDSLSISLVTVVLSAVIYPIFLRSMVWGL
NCU06776.1	VAKPSEKAVRNNNSDVGTLMKKFWGEMPSIFLQSLNQTLASLILTLVTYP-FLRKVVWRL
FG10865.1 	LSKG-------KNEKQPDALKLVYQAIPHTFSDSVAGAAAALPVALILYYAILRSFIWGW
AN4417.1  	ITDKPVGGADQKTHPTQPISKRLQEGLHQTLRDGLFRSTVVALVCPVAYVFFLRRRAWTY
          	::. .               * .   :   : :.:  : .   :  : *  :**   *  

MG03886.1 	ALSMFRPFYSITHVNLVPP--TLPFTFWLLLRCISSTFMLSFIWLSGNTAFSILLTRDPI
NCU06776.1	SLFFFRPFFNLPKTNYVPY--SWPVSFKSATSIILASTMLLIVWIAGNTAFTMFMVKEPT
FG10865.1 	ALMFLRIFYNLPKTNMLPP--SWPSDLWLLFRCMEAGTLIHLIWNIGNFAFSSFMVKAPL
AN4417.1  	TMSFAKLFWDFPRSAADPPSLILPVKPPLIARTIFSGALLVLCWQTTNLFFSMFLSQEPV
          	:: : : *:.:.:    * :   *         : :  :: : *   *  *: :: : * 

MG03886.1 	KNTKPLTSDSKDPNGSLLNGLKSKKLPVK0CFAMWELGLIACDFDVRRKAIYEDIDRKDG
NCU06776.1	KDQRPFTSGSKDPNGSLLNGLGHRKLFYK0CFAMWELALIARDFPERRKAIYEDLDRKDG
FG10865.1 	KNGKPLTSESKDPNSSLLNGLKSKKLSIK0AFAMWELAYIAQGFDTRRQAIYEDIDRKDG
AN4417.1  	KRGQPLTSGAKDPTGSLLNGLKAKKETVR~AFAFWELCFISQQFPDRRKAIFNDIDREGG
          	*  :*:** :***..******  :*   : .**:***  *:  *  **:**::*:**:.*

MG03886.1 	PVWAQVFRVCLEVIKEMETNIDNYGKVP-TPAPATEANSSADGRFSEEVQRGIQAPKNES
NCU06776.1	PAWSQVCSICLEVLKSLETNIGNYGKAP-EPVAAPVVQP------VEEKVRTAPPPKEEP
FG10865.1 	PMWSQVYSICLDVIKSIETRVDTYGKAPDAPLAAPAAEP---------KQRVSTPLRDDP
AN4417.1  	SAWTQIMQSATEVIQGITSRINEQKLGPAKSKPSPAETKSEP--TLRTLPRLTDPVKEGN
          	. *:*:   . :*:: : :.:.     *  . .:.    :          *   . ::  

MG03886.1 	IEIK--SSKNAFRAEVEKYVDKLARDPSR----ASRLDDVGK-AAGEAKQKLLEMRN---
NCU06776.1	IFLGGPARRRNFLSEVEKTVVEVAVAPGQ----GSPLKPFAKRVIDAAKHQLLEIQK---
FG10865.1 	IFNN-RAAPRTMRSEVEKRLSQVARSPGESP--ASKLSPIAKKTWKEAKDRVLTKEQQ--
AN4417.1  	IFAA-SPRSTTTNGRLGEGFSSTLRSLGESPDWTPKARARARDVFDRASSAVLSPERKQK
          	*     .      ..: : . .     ..:..  .     .: .   *.  :*  .....

MG03886.1 	---------QLAVSDDPQYPFQQYIRNILSS-PVGWPLRQDYSRRLTAAVFGAPYGEPSL
NCU06776.1	---------AVTGTDDTASLIRSWALKGLHK-WVGIPFRKEYRRRMLHAVLGSPYGEPSL
FG10865.1 	---------ELVTPEHLKGQFEQWTLQLMEVDAIAALFQQRFRTQFAAAVLGTPYAEPTL
AN4417.1  	LLSSSQDFKLLTGASSTRPESIHPFLAQLLRSPIGLLIRQPFARRLSGIVLGTPTASLSS
          	  :::.. . :. ..             :  . :.  ::: :  ::   *:*:* .. : 

MG03886.1 	FVNATVALSQLAVRSLQEDKYGNVQRDVATIIRSLTSVTQKLDTFK-NSLPFHWTDVQK-
NCU06776.1	YINAAYALSMLTSHSLQEDKYGNVQRDIATIIRTLTNMTKKLDRFK-EGFEVHWTDVEG-
FG10865.1 	CINAIHVLHHLSVHSLTEDQFGNVHRDVPGIIRTFTSVIKKLEAFR-IEFPLHWTDVSC-
AN4417.1  	IIDAIESITRLLIASLNEDPYGKVQADVPAIVSLFTETLTTLDTYAHVELDVHWTDAAFP
          	 ::*   :  *   ** ** :*:*: *:. *:  :*.   .*: :    : .****.  .

MG03886.1 	----------HRSCPEVEIVLEALRDGLSELVLNFEPYRRDLRLSLTDMRLAREAIGVAE
NCU06776.1	----------IKECPEVDAILEALREALTTLIVEFGPYARDLRLTLTDMRLAREAANFPE
FG10865.1 	----------KRVSPEVEKVMDALKTALKQVLANFEPYSSDLRLTLSDIRLAKEACADAK
AN4417.1  	PSNDPAAQAAARRVPDVELIIDSLRASLKSLLTAFSPYFKDVGLVGKELRLAKGAAGFDE
          	.:...::.:: :  *:*: ::::*: .*. ::  * **  *: *  .::***: *    :

MG03886.1 	TAVEPTPQAKRAAKPPAPEMQELR
NCU06776.1	----PQPRADHLLDLNQWKR----
FG10865.1 	-----NSEMTQVQS----------
AN4417.1  	E-----------------------
          	
```
